# Hypomethylation of 111 Probes Predicts Poor Prognosis for Glioblastoma

**DOI:** 10.3389/fnins.2019.01137

**Published:** 2019-10-25

**Authors:** Qi Chen, Min Zhao, Chengliang Yin, Shiyu Feng, Jian Hu, Qiang Zhang, Xiaodong Ma, Wanguo Xue, Jinlong Shi

**Affiliations:** ^1^National Engineering Laboratory for Medical Big Data Application Technology, Chinese PLA General Hospital, Beijing, China; ^2^Medical Big Data Center, Chinese PLA General Hospital, Beijing, China; ^3^Department of Neurosurgery, Chinese PLA General Hospital, Beijing, China; ^4^Department of Cancer Biology, The University of Texas MD Anderson Cancer Center, Houston, TX, United States; ^5^Department of Biomedical Engineering, Chinese PLA General Hospital, Beijing, China

**Keywords:** glioblastoma, biomarker, methylation, joint-score, prognostic evaluation

## Abstract

Glioblastoma (GBM) is a complicated brain tumor with heterogeneous outcome. Identification of effective biomarkers is an urgent need for the treatment decision-making and precise evaluation of prognosis. Based on a relatively large dataset of genome-wide methylation (138 glioblastoma patients), a joint-score of 111 methyl-probes was found to be of statistical significance for prognostic evaluation. Low joint-score were significantly associated with adverse outcomes (OS: *P* < 0.001, PFS: *P* = 0.03). Multivariable analyses adjusted for known risk factors confirmed the low joint-score of 111 methyl-probes as a high risk factor. The prognostic value of the methylated joint-score was further validated in another dataset of glioblastoma patients (OS: *P* = 0.006). Additionally, variance analysis revealed that aberrant genetic and epigenetic alterations were significantly associated with the joint-score of those methyl-probes. In conclusion, our results supported the joint-score of 111 methyl-probes as a potential prognosticator for the precision treatment of glioblastoma.

## Introduction

Glioblastoma (GBM) is by far the most aggressive and infiltrative type of brain tumors with great hazard rating and poor prognosis, which accounts for more than half of all gliomas (Nabors et al., 2018). The progress of identifying novel biomarkers has significantly improved the precision diagnosis and treatment of glioblastoma. A brain tumor which contains isocitrate dehydrogenase (IDH) and telomerase reverse transcriptase (TERT) mutation, accompanied with 1p/19q deletion would be diagnosed as an oligodendroglioma; IDH mutation with wild TERT is a characteristic of astrocytoma (Labussiere et al., 2010; Sahm et al., 2014). Both IDH1 and IDH2 mutation were favorable prognostic factors of lower-grade gliomas distinguished from primary glioblastomas (Sanson et al., 2009). However, H3 histone family member 3A (H3F3A) mutation is evidence of infiltrative glioma and an adverse prognostic marker before GBM establishment (Sturm et al., 2012; Meyronet et al., 2017). Aberrant methylations of *MGMT*, *TERT*, and *EGFR* have been reported to have potential value in diagnosing gliomas (Bady et al., 2012; Kros et al., 2015; Arita et al., 2016). Besides, O-6-methylguanine-DNA methyltransferase (*MGMT)* promoter methylation status is devoted to promote the therapeutic effect of temozolomide on GBM (Hegi et al., 2005; Wick et al., 2012). Temozolomide, as an alkylating agent, could damage the DNA and trigger the death of tumor cells (Nabors et al., 2018). A combined expression signature of nine genes was reported to be helpful for predicting the outcome of glioblastoma patients receiving temozolomide therapy (Colman et al., 2010). Genome-wide assessments of cancer epigenome (array and sequencing technologies) enable to find new combined signatures as clinical biomarkers, which could promote diagnostic decision and also reveal the complex tumor mechanisms. Glioma-CpG island methylator phenotype (G-CIMP) defines a proneural subgroup of lower-grade glioma patients with younger diagnosis age and better outcomes (Bady et al., 2012; Eckel-Passow et al., 2015). A nine-gene methylation signature had implications in evaluating abnormal NF-kB signaling of GBMs (Shukla et al., 2013). Besides, a three-CpG panel at non-CpG island regions and a six-CpG panel from genome-wide methylation were two newly developed prognostic indicators for GBMs (Yin et al., 2017; Yin et al., 2018). However, platform heterogeneity of the data sources and excluding none-CpG probes limit the applicability of these signatures to small subgroups of GBMs.

In this study, via integrating genome-wide DNA methylation data and clinical information, we reported a novel biologically relevant methyl-probe panel for rapid risk stratification of GBMs. The signature robustly predicts survival risk of GBM patients in a treatment-independent manner and is of promising value to improve current patient management.

## Materials and Methods

### Patient Cohorts

Available GBM datasets with DNA methylation data measured from brain tissue were systematically collected from main public repositories [The Cancer Genome Atlas (TCGA), NCBI Gene Expression Omnibus (GEO) repository, GEOmetadb and ArrayExpress], more than 600 samples with clinical information were obtained. In order to eliminate platform heterogeneity, only those data derived from Illumina Human Methylation 450 array (GPL13534) were kept in this study. Patients with an overall survival (OS) time ≥0.5 month were kept for survival analysis. At last we achieved two satisfactory datasets as TCGA_450k (*n* = 138, median age is 60.77 years) and GSE60274_450k (*n* = 62, median age is 51.5 years). Furthermore, 97 samples in the TCGA_450k cohort have clinical indicator information about MGMT, G_CIMP, IDH1, and Treatment strategy (Supplementary Table S1), based on which the Cox proportional hazards regression analysis was carried out for the newly identified methyl-probe signature.

### Filtering Process of Methyl-Probe Signature

Illumina Human Methylation 450 array contains 485,577 probes, which are derived from different genomic regions of 9,988 genes. Methylation level of each probe was represented as β value, ranging from 0 (completely unmethylated) to 1 (completely methylated). Preprocessing was performed on the TCGA_450k cohort to eliminate probes with null value, and 382,452 probes with methylation level (0 < β < 1) were kept for downstream analysis. Prognostic signatures were constructed via a series of processes. Firstly, univariate Cox proportional hazards regression analysis was carried out for each probe in the TCGA_450k cohort. Significant methylation probes were chosen with the adjusted *P*-value cutoff (*P* < 0.05, adjusted by false discovery rate). 35,708 probes without null values were kept. Secondly, 62 known genes associated with glioma were found in both tumor gene list of Catalogue of Somatic Mutations in Cancer (COSMIC) and glioma gene list of Online Mendelian Inheritance in Man (OMIM) (Supplementary Table S1 – Sheet S3). 4,366 out of 5,390 probes for the 62 genes were without null value in TCGA_450k cohort and used. Thirdly, the overlapping of significant methylation-and 62 gene-related probes were selected, and dispersion statistic with boxplot method was created based on the hazard ratio (HR), outliers that lie beyond the extremes of the whiskers were excluded. Finally, the HRs were used to build the percent weighted coefficients, and probes with weight coefficients over 0.001 were kept to construct survival risk classification model, which was the methyl-probe signature with 111 probes. The median of the methyl-probe score from the training dataset, TCGA_450k, was set as the cutpoint for stratifying high- and low-risk GBM tumors. Validation phase was performed in both TCGA_450k cohort and an independent cohort of GSE60274_450k.

### Statistical Analysis

All statistics were performed in R (version 3.4.4). Survival package (version 2.42) (Therneau and Grambsch, 2000) was used for survival risk comparison. Chi-square test was used to evaluate the relationship between methyl-probe and Gender, MGMT, G_CIMP, IDH1, and Treatment. Two-sided *t*-test was performed for age, PFS_day and os_month. Univariate and multivariate Cox proportional hazards regression were tested on Age, Gender, MGMT status, G-CIMP status, IDH1 mutation, Treatment, Three-CpG panel, Six-CpG panel, and Methyl probe signature. Descriptive statistics with boxplot method was carried out for eliminating outliers of methylation probes. Discovery of significant differential methylation probes was based on the β value of Illumina Human Methylation 450 array by Empirical Bayes (Limma package, version 3.34) between high- and low-risk GBM tumor groups. 1,768 genes of the 3,000 most significant methylation probes (Supplementary Table S3) were determined by the Illumina Human Methylation 450 platform information, and GO analysis was carried out by MetaScape^1^.

## Results

### Identification and Construction of the Joint-Score of Methyl-Probes

**FIGURE 1:**
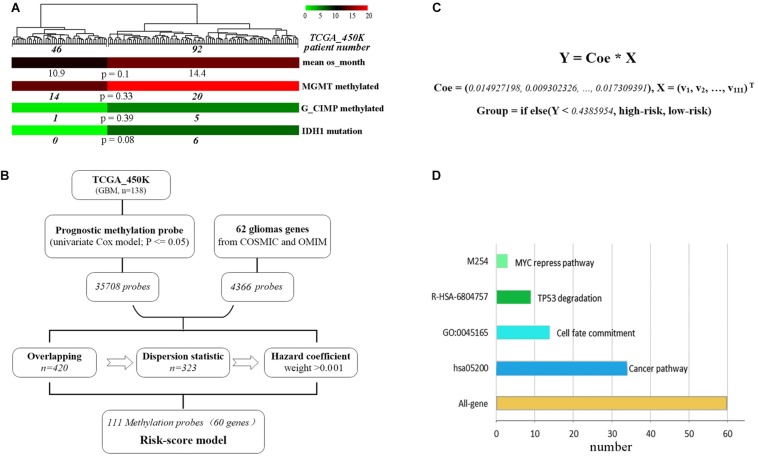
The development of the methylation risk-score signature. **(A)** GBM clustering by the methylation probes of 62 glioma-tumor genes. **(B)** Study workflow for the construction of joint-score signature. **(C)** The joint-score formula constructed with methyl-probe panel. X: β value of the optimal methylated probes. **(D)** Characteristics of biological function for methyl-probe corresponding genes.

A total of 62 glioma-tumor related genes were identified and their methylation probes were used for clustering 138 GBM patients. The group of 46 patients with shorter OS, had a non-significant tendency in lower promoter methylation of MGMT or G_CIMP, and a trend but not significant in fewer mutations of IDH1 (Figure 1A). This indicated that methylation loci had the potential to act as effective biomarkers to evaluate the prognosis for GBMs.

To generate the optimal representative methylation probes, genome-wide methylation arrays were used. 449,869 probes with none significant hazard (*P* > 0.05) were filtrated after univariate Cox analysis, and 35,708 prognostic methylation probes were overlapped with 4,366 ones associated with those 62 glioma-tumor genes, the remained probes were used for further odd probe elimination (Figure 1B). The per centum of hazard value for each remained probes of TCGA_450k was calculated after boxplot filtration. Only the weight value >0.001 were left as prognostic coefficient, and 111 methyl-probes constituted the joint-score formula with the optimal cutoff of 0.4386 (the median risk value from the discovery cohorts) for stratifying low-risk and high-risk patients (Figure 1C, Supplementary Figure S1, and Supplementary Table S2). The methyl-probes covered 60 glioma-tumor genes, 34 of which were involved in the cancer pathway and 20 genes participated in the process of Cell fate or TP53/MYC regulation signaling (Figure 1D and Supplementary Table S2).

### The Prognostic Value of the Methyl-Probe Signature

**FIGURE 2:**
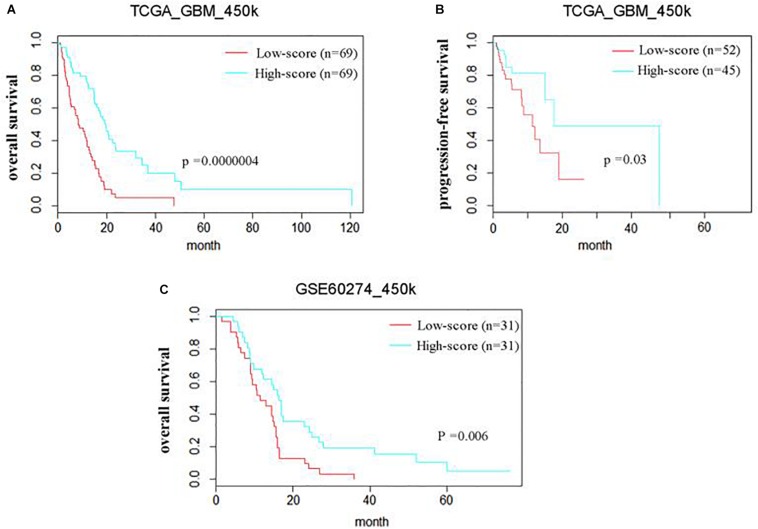
The methyl-probe signatures have significant prognostic value in independent groups of glioblastoma patients. **(A,B)** Risk classification of Methyl-probe signature in a pooled survival analysis of TCGA_450k. *P*-values from meta-analysis <0.001 for pairwise comparison. **(C)** Risk classification using Methyl-probe signature in a pooled survival analysis of GSE60274_450k. *P*-values from meta-analysis <0.01 for pairwise comparison.

The prognostic efficiency of the 111 methyl-probes was validated for GBM risk stratification. Patients were divided into high-risk group (with low joint-score, *n* = 69) and low-risk group (with high joint-score, *n* = 69) in the discovery cohort of TCGA_450k, where high-risk patients were consistently associated with shorter OS and progression-free survival (PFS) than low-risk ones (Figures 2A,B and Table 1). Besides, well-known molecular marks, G_CIMP and IDH1, showed certain interrelationships with the methyl probe signature. And the unmethylated G_CIMP and wild IDH1 tumors contributed to low methyl score and relative high prognostic risk. Compared to these molecular markers in NCCN guideline (G_CIMP and IDH1) and previous reported prognostic signatures, the 111 methyl-probes showed more efficiency in glioblastomas risk clarification (Supplementary Figure S2). The joint signature was further tested in an independent cohort of heterogeneous population, GSE60274_450k, and it accurately stratified high-risk and low-risk patients with chemotherapy or radiation or combined treatments (Figure 2C), indicated its generality of predicting power for GBM-specific survival progression.

**TABLE 1:** Molecular and clinical characteristics for the discovery set of TCGA_450K GBMs.

	**Methyl-probe signature**			**TCGA_450k GBM**
		
	**Low-score**	**High-score**	***P*-value**	
Sample size	69	69		138
**Age**			**0.027**	
Median	63.9	59		60.77
Range	23.4–85.6	21.4–77.9		21.4–85.6
**PFS_day**			0.135	
Median	146	167		157
Range	29–797	0–1,458		0–1,458
**os_month**			**<0.001**	
Median	6.7	14.9		9.1
Mean	8.9	17.5		13.2
**Gender**			0.955	
Male	30	25		55
Female	22	19		41
**MGMT**			0.303	
Methylated	15	19		34
Unmethylated	28	16		44
**G_CIMP**			**0.008**	
+	0	6		6
−	52	39		91
**IDH1**			**0.008**	
Mutation	0	6		6
Wild	50	37		87
**Treatment**			0.248	
chem	20	24		44
RT	14	6		20
RT/chem	16	11		27

*G-CIMP, glioma-CpG island methylator phenotype; RT, radiotherapy; Chem, temozolomide or alkylating chemotherapy. The bold values in left column are analytical factors, and the *P*-values below 0.05 are in bold for marking the significant ones.*

### The Methyl-Probe Signature Was an Independent and Superior Prognostic Factor for GBMs

Patient characteristics of stratified cohorts supported that the methyl-probe signature was not only a predictive indicator for part of the clinical index, but also a prognostic factor for GBMs (Table 1). Within TCGA_450k samples, univariate Cox regression model revealed that age, IDH mutation status, Three-CpG panel, and the methyl-probe signature were significantly correlated with OS (Table 2). MGMT promoter and G-CIMP methylation status showed the risk effect but not significant. Meanwhile, the six-CpG panel failed to show risk relation to the TCGA_450k GBM tumors. Multivariate Cox model further demonstrated that the methyl-probe signature was an independent and protective prognostic indicator (Table 2).

**TABLE 2:** Results for Cox regression models of the methyl-probe signature.

**Variables**	**Univariate Cox model**			**Multivariate Cox model^a^**		
		
	**HR**	**95% CI**	***P*-value**	**HR**	**95% CI**	***P*-value**
Patient Age	1.043	1.021–1.066	**<0.001**	1.029	1.004–1.055	**0.024**
Gender	0.8295	0.514–1.338	0.444			
MGMT status	1.519	0.881–2.618	0.133			
G-CIMP status	5.403	0.749–38.97	0.094			
IDH1 mutation status	4.456	1.074–18.48	**0.039**	0.234	0.029–1.849	0.167
Treatment	1.139	0.899–1.441	0.281			
Three-CpG panel	1.134	1.002–1.284	**0.046**	1.063	0.889–1.271	0.505
Six-CpG panel	1.097	0.955–1.26	0.192			
Methyl-probe signature	0.001	3E-05–0.018	**<0.001**	2.47E-05	6.8E-08–0.0089	**<0.001**

*HR, hazard ratio; CI, confidence interval; RT, radiotherapy; G-CIMP, glioma-CpG island methylator phenotype; Treatment, Chem vs. RT vs. Chem/RT; Chem, temozolomide or alkylating chemotherapy. In bold are significant results for Cox regression models. ^*a*^Multivariate Cox regression analysis was performed by incorporating significant factors in the univariate Cox model.*

### Molecular Characterization Associated With the Methyl-Probe Signature

**FIGURE 3:**
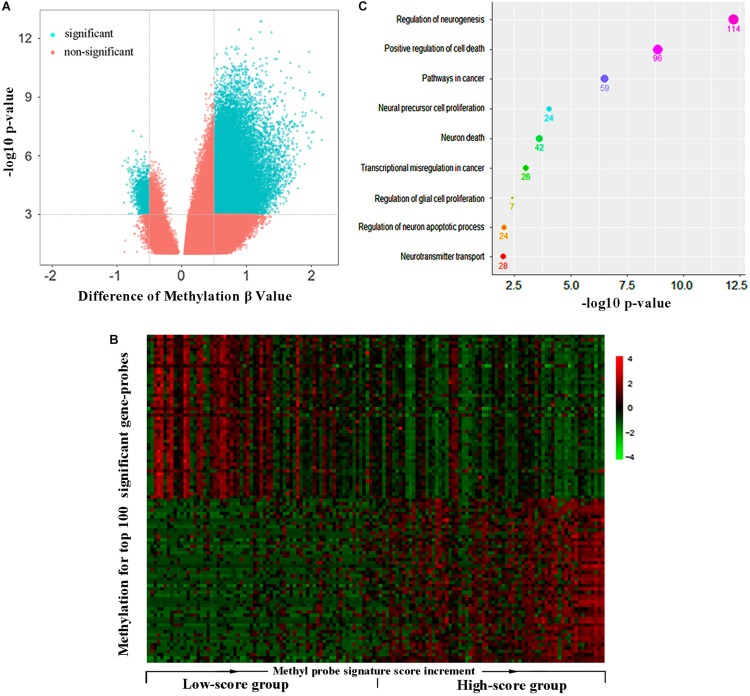
Methyl-probe characteristics between high- and low-score GBM tumors. **(A)** Volcano plot for significantly differential methylation probes between high- and low-risk GBM tumors. Blue, significant probes. **(B)** Hierarchical clustering of methylation profiles highlights the differences between the low and high joint-score tumors. Gene probes of significantly methylation difference between the low and high joint-score tumors are sorted into a queue by decreasing their *P*-value, and 50 probes at each head or end of the queue are presented in the plot. **(C)** Enrichment characteristics in cancer and neural associated pathways for the significant methyl-probe corresponding genes. Count under the dot, the gene number belonging to the biological pathway.

All the GBM patients were divided into high- and low-score groups according to the median of methyl-probe joint-score, and differential methylation analysis was carried out between these two groups. Results demonstrated that 8,361 out of total 9,988 genes covered by Illumina Human Methylation 450 array were in differential methylation status, and 8,153 differential genes were in hypermethylation status in the high-score signature group (Figures 3A,B). In the 111 methyl probes, 45 genes black colored in Supplementary Table S2, were in the significant hypermethylation status, which implied that the methyl-probe signature was greatly relevant to the major subgroup of methylation differential genes. The low-score signature group with high hazard risk significantly had overall low level of methylation in most methylation differential genes, which implied that hypomethylation of the methyl-probe signature accompanied with relatively high risk in GBMs. Gene ontology analyses performed on 1,768 genes from 3,000 methylation differential probes between high and low risk GBM groups. Among these genes, NGFR, PRKN, STAT3, IDH2, etc. were associated with the neuronal system and cancer pathway as the significant terms (*P* < 0.001) (Figure 3C and Supplementary Table S3). This indicated that methyl-probe signatures have great potential to play as new biomarkers in clinical diagnosis and prognostic evaluations for GBM.

## Discussion

In this report, we incorporated genome-wide DNA methylation data and clinical information to generate a joint-score of 111 methyl-probes, which could be used as a significant biomarker to evaluate survival risk for glioblastomas. All the 111 methylation probes are derived from the same platform (Illumina Human Methylation 450 array), which guarantees a convenient usage in making the patients’ prognosis demarcated, which has a great potential to make simple detection kit and refine the GBM risk classification.

There are several signatures of methylation on molecular and clinical grounds developed for characterizing human gliomas and helping make treatment decisions (Wang et al., 2018; Widschwendter et al., 2018). Methylated MGMT promoter particularly benefits the elderly glioblastoma patients in treatment with temozolomide than those none-methylated ones (Thompson et al., 2010). Methylation of G-CIMP was prevalent among lower-grade gliomas and accompanied with improved outcome in the proneural subgroup (Noushmehr et al., 2010). Recently, a hypomethylation signature with three-CpG at none-CpG island regions was identified as a poor prognosis indicator for GBMs (Yin et al., 2017). Besides, a six-CpG signature based on MGMT and G-CIMP methylation status robustly predicted OS of gliomas in a treatment-independent manner (Yin et al., 2018). However, the limitation still existed in their clinical application because their effectiveness just covered a small subset of patients, which might due to the platform heterogeneity between the methylation array 27K and 450K or the incomplete genome wide CpG loci.

In the methylation difference analysis, 96% of marked methylation loci in GBM group with high-score methylation signature were in hypermethylation status, the same trends about 98% were also founded in another independent study (Noushmehr et al., 2010). Epigenetics controls expression potential, rather than expression state (Gyorffy et al., 2016). A previous study reported that a total of 300 genes were with significant changes of both DNA hypermethylation and gene expression in two subgroup of glioma, in which 263, about 87.7%, were downregulated and hypermethylated (Noushmehr et al., 2010). In the significant differential methylated genes between the low and high joint-score tumors, genes like NGFR and NrCAM, were identified in independent analysis to be highly prognostic in head carcinoma (Sakurai, 2012; Berghoff et al., 2015; Ahn et al., 2016).

In summary, the joint-score of 111 methyl-probes is an independent prognostic biomarker, and has implications for differential therapeutic strategies for glioma patients. Although this work requires further validation, the novel methylation signature and relevant gene network may provide new insights into prognostic classification, molecular characterization, and treatment development for GBMs.

## Author Contributions

JS, WX, and XM designed the study. QC performed the study and analyzed the data. QC and JS wrote the manuscript. SF, JH, and QZ provided the expert consultations and clinical suggestions. All authors reviewed the final version of the manuscript.

## Conflict of Interest

The authors declare that the research was conducted in the absence of any commercial or financial relationships that could be construed as a potential conflict of interest.

## Footnotes

1 http://metascape.org/gp/index.html
