# Supplementary figures and images for: Hypomethylation of 111 Probes Predicts Poor Prognosis for Glioblastoma
Source: Front Neurosci. 2019 Oct 25;13:1137. doi: 10.3389/fnins.2019.01137 (PMC6823878; doi:10.3389/fnins.2019.01137)

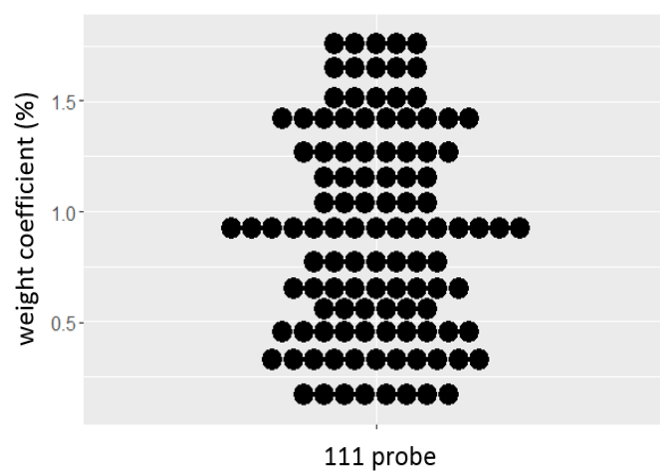

Fig. S1. Contribution degree of each probe of the 111 methyl-probe signature

Supplement: Supplementary file 1 [file Data_Sheet_1.PDF]
