# Supplementary material for: Hypomethylation of 111 Probes Predicts Poor Prognosis for Glioblastoma
Source: Front Neurosci. 2019 Oct 25;13:1137. doi: 10.3389/fnins.2019.01137 (PMC6823878; doi:10.3389/fnins.2019.01137)
Supplement: Supplementary file 2 [file Data_Sheet_2.PDF]

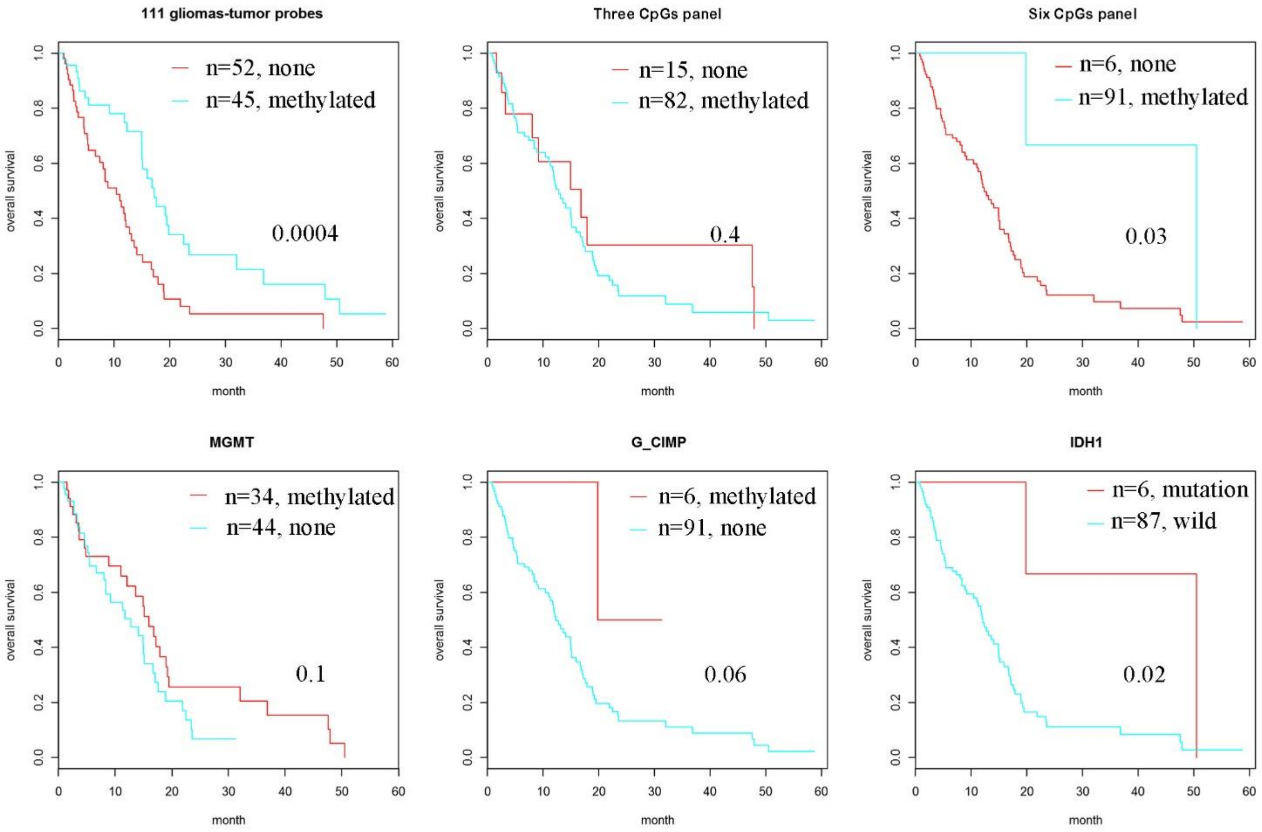

Fig. S2. Prognostic capability comparison of the molecules in glioblastoma patients. Survival analysis of TCGA_450k GBM dataset was performed for risk classification of each signature, 111 methyl-probe signature, three CpGs panel, six CpGs panel, MGMT methylation, G_CIMP methylation, IDH1 mutation.
